# Scour Damage Detection and Structural Health Monitoring of a Laboratory-Scaled Bridge Using a Vibration Energy Harvesting Device

**DOI:** 10.3390/s19112572

**Published:** 2019-06-06

**Authors:** Paul C. Fitzgerald, Abdollah Malekjafarian, Basuraj Bhowmik, Luke J. Prendergast, Paul Cahill, Chul-Woo Kim, Budhaditya Hazra, Vikram Pakrashi, Eugene J. OBrien

**Affiliations:** 1 School of Civil Engineering, University College Dublin, D04V1W8 Dublin, Ireland; paul.fitzgerald.3@ucdconnect.ie (P.C.F.); abdollah.malekjafarian@ucd.ie (A.M.); eugene.obrien@ucd.ie (E.J.O.); 2 Dynamical Systems and Risk Laboratory, School of Mechanical and Materials Engineering and Centre for Marine and Renewable Energy Ireland, University College Dublin, D04V1W8 Dublin, Ireland; basuraj.bhowmik@ucd.ie (B.B.); paul.cahill@ucd.ie (P.C.); 3 Department of Civil Engineering, Faculty of Engineering, University of Nottingham, Nottingham NG7 2RD, UK; luke.prendergast@nottingham.ac.uk; 4 Department of Civil and Earth Resources Engineering, Kyoto University, Kyoto 615-8540, Japan; kim.chulwoo.5u@kyoto-u.ac.jp; 5 Department of Civil Engineering, Indian Institute of Technology, Guwahati, Assam 781039, India; budhaditya.hazra@iitg.ac.in

**Keywords:** bridge, scour, energy harvesting, damage detection, structural health monitoring, singular spectrum analysis, frequency shifts

## Abstract

A vibration-based bridge scour detection procedure using a cantilever-based piezoelectric energy harvesting device (EHD) is proposed here. This has an advantage over an accelerometer-based method in that potentially, the requirement for a power source can be negated with the only power requirement being the storage and/or transmission of the data. Ideally, this source of power could be fulfilled by the EHD itself, although much research is currently being done to explore this. The open-circuit EHD voltage is used here to detect bridge frequency shifts arising due to scour. Using one EHD attached to the central bridge pier, both scour at the pier of installation and scour at another bridge pier can be detected from the EHD voltage generated during the bridge free-vibration stage, while the harvester is attached to a healthy pier. The method would work best with an initial modal analysis of the bridge structure in order to identify frequencies that may be sensitive to scour. Frequency components corresponding to harmonic loading and electrical interference arising from experiments are removed using the filter bank property of singular spectrum analysis (SSA). These frequencies can then be monitored by using harvested voltage from the energy harvesting device and successfully utilised towards structural health monitoring of a model bridge affected by scour.

## 1. Introduction

Bridge scour refers to the excavation of soil from around a bridge foundation by hydraulic action and is the most common cause of bridge collapse worldwide [1]. It causes a decrease in soil elevation relative to the bridge foundation and this compromises the structural integrity of the bridge. For the cases of bridges founded on shallow pads, scour can reduce the soil-structure contact area. As a result, increased stresses occur in the remaining soil area, which leads to increased soil strains. This causes the shear stiffness of the foundation system to be compromised, which can lead to adverse settlements.

The consequential reduction in stiffness has led to the field of vibration-based scour monitoring which is based primarily on monitoring changes in modal properties (frequencies and mode shapes) incurred as a result of this stiffness loss [2,3,4,5]. This scour detection process generally entails the installation of sensors on the structure (e.g., accelerometers) so that these modal properties can be monitored. Thus far, research has been carried out under this premise in both numerical and experimental studies. 

Prendergast et al. [6,7] numerically investigate the case of a scoured central pier of a two-span integral bridge and use changes in the first natural frequency as a means of scour detection. The robustness of the method has been demonstrated by taking into account the influence of parameters such as vehicle variations (speed and mass) and sensor noise on the resulting lateral pier vibrations. An extension of the method is demonstrated in reference [8] and multiple bridge frequencies are used to locate the scoured pier or abutment. Ju [9] investigates the effects of water-added foundation mass on the natural frequency of a bridge under scour and concludes that a frequency reduction occurs due to scour, with a lower corresponding frequency when water-added mass is considered. Klinga and Alipour [10] numerically investigate the scenarios of extreme scour at various bridge elements and show that the lateral stiffness and natural frequency are reduced as a consequence of scour. 

Experimental vibration-based studies have been carried out on both full-scale and laboratory scaled bridges. A five-span bridge where one pier has experienced scour is studied by Foti and Sabia [11]. Asymmetric dynamic behaviour of a pier as a result of uneven pier scour is monitored and it is concluded that the presence of scour is detectable. A laboratory scaled coastal bridge model is used by Elsaid and Seracino [4] to investigate the effects of scour. Here, scour is modelled as increased length of exposed pile and it is demonstrated that the horizontally displaced mode shapes are influenced by scour. Chen et al. [12] apply a vibration-based approach to a cable-stayed bridge and use ambient velocity measurements in conjunction with finite-element updating to detect scour. Xiong et al. [13] also examine scour detection of a cable-stayed bridge and recommend a flexibility-based deflection approach as a viable scour indicator. 

The growth of vibration-based structural health monitoring (SHM), not just limited to bridge scour applications, has led many researchers to investigate the feasibility of replacing wired sensing technology with a network of wireless sensors [14,15]. This is motivated by the great cost of wiring between sensors and data acquisition systems [16]. However, a significant challenge remains around the issue of providing an electrical power source to such devices. Park et al. deploy a wireless smart sensor network SHM system on a cable-stayed bridge and propose the use of energy harvesting devices (EHDs) or the use of self-powered sensor nodes to address the issue [17]. Vibration-based EHDs can use ambient vibrations of a host structure to produce a feasible source of power for such sensor nodes [18]. Suitable proposed host structures for such EHDs include high-rise buildings [19] and tunnels [20] but the majority of studies thus far investigate the use of bridge structures as a host [21]. Piezoelectric EHDs are one such device and have the potential to harvest energy using operational bridge conditions, typically using the forced vibration bridge response due to vehicle passages [16,22,23]. 

Instead of using the harvested energy to power sensor nodes, the use of the harvester as a direct SHM device is a research topic in its infancy [23]. Here, the electrical signal output itself is used as an SHM tool. This may entail extracting bridge dynamic features (frequencies, mode shapes etc.) from the harvester outputs or using changes in the harvester outputs itself (e.g., power) to infer abnormal changes related to structural defects in the host structure. Cahill et al. [22] experimentally demonstrate that the power of a piezoelectric beam-hosted EHD increases for the situation of a two-axle model vehicle crossing a beam with a crack and further increases are recorded with an increase in crack severity. In a separate experimental work, Cahill et al. [23] extract bridge frequency information from a piezoelectric cantilever-based EHD attached to an operational bridge undergoing forced vibration from a train passage. 

This work examines the potential to use a cantilever-based piezoelectric EHD as an SHM tool for the detection of scour on a bridge with multiple simple spans. An experimental scaled bridge model consisting of four simple spans is created and each pier is supported on springs. This allows for the modelling of scour by reducing the spring stiffness under a pier to model the loss of stiffness that would result from scour. Here, it is demonstrated how the frequencies of the bridge can be extracted from the raw EHD output voltage generated during the bridge free-vibration stage after a vehicle crossing. In this case, the harvester is installed at the central bridge pier. It is subsequently shown how changes in these frequencies can be detected from the EHD voltage. Furthermore, it is demonstrated that the EHD does not need to be located at a scoured pier, as it can detect changes resulting from scour at another pier. This is because the observed frequency shifts are related to changes in the global modes of the bridge meaning that the number of EHDs may in fact be less than the number of supports in a bridge with multiple simple spans. Using the inputs from a single EHD, the harmonic loading and electrical interference inevitably arising as noise components during experimentation are removed using the filter bank property of singular spectrum analysis (SSA). The significant results obtained from this, based on the filtered frequency components, suggest that EHDs may have the potential to be used as stand-alone devices in the vibration-based bridge scour detection field. 

## 2. Piezoelectric Energy Harvesting Device Description

**Figure 1:**
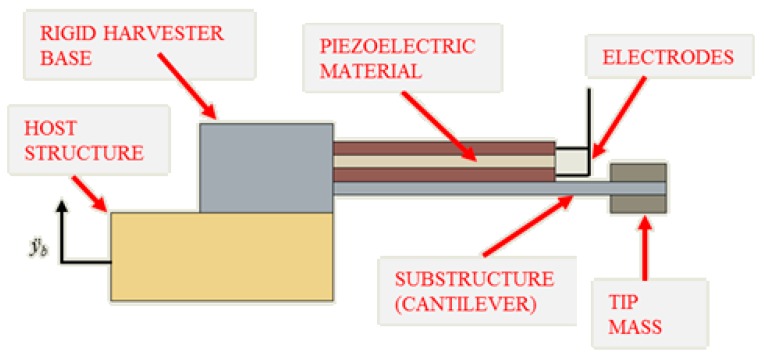
Piezoelectric cantilever-based harvester with base excitation ${\ddot{y}}_{b}$.

Piezoelectric energy-harvesting devices (EHDs) convert strain fluctuations in the piezoelectric material to electrical energy. Figure 1 shows a cantilever-based EHD and is the device used in this work. It consists of piezoelectric material attached to a cantilever substrate and the piezoelectric material has bound electrodes which allow for the generated electrical energy to be availed of. The cantilever is clamped into a rigid base, which in turn is attached to a host structure. The acceleration response of the host structure, ${\ddot{y}}_{b}$, acts as a base excitation source for the harvester. At the free-end of the cantilever is a tip mass which often is adjustable to allow for frequency tuning of the device [24].

The electromechanical behaviour of a piezoelectric EHD can be represented by the two linearly coupled Ordinary Differential Equations (ODEs) [16,21,25]

(1) $$m_{h}\ddot{z}+c_{h}\dot{z}+k_{h}z-\theta V=-m_{h}{\ddot{y}}_{b}$$

(2) $$\theta \dot{z}+C_{p}\dot{V}+\frac{1}{R_{l}}V=0$$

where $m_{h}$, $c_{h}$, and $k_{h}$ are the mass, damping and stiffness of the harvester respectively; z is the relative dynamic displacement of the tip mass $m_{h}$, with over-dots referring to differentiation with respect to time; $y_{b}$ is the base excitation of the harvester and θ, V, $C_{p}$ and $R_{l}$ refer to the electromechanical coupling, voltage generated, piezoceramic capacitance and load resistance, respectively. The natural frequency of the harvester in units of $\mathrm{rad}\;s^{-1}$, $\omega_{h}$, is defined as

(3) $$\omega_{h}=\sqrt{\frac{k_{h}}{m_{h}}}$$

and the natural frequency in units of Hz is denoted using the symbol $f_{h}$ in this work. The harvester damping, $c_{h}$, can be represented as $c_{h}\;=\;2\xi_{h}m_{h}\omega_{h}$, with $\xi_{h}$ being the damping ratio. The nondimensional time constant of the first-order electrical system, α, is defined as $\alpha \;=\;\omega_{h}C_{p}R_{l}$ [16]. On examination of Equation (3) it is clear that the voltage produced by the energy harvester, V, is related to the base excitation of the structure, ${\ddot{y}}_{b}$. Due to this, it is expected that the frequency content of the excitation source is contained in the harvested voltage. To demonstrate this, a simple numerical harvester simulation is conducted, and the generated harvester voltage is examined in the frequency domain. For this simulation, the base excitation source is selected to be

(4) $${\ddot{y}}_{b}=Ae^{-\xi_{b}2\pi f_{b}t}\sin(2\pi f_{b}t)$$

where *A*, $\xi_{b}$ and $f_{b}$ are arbitrarily chosen to be $2\;{\mathrm{ms}}^{-2}$, 0.04 and 7 Hz respectively. The harvester parameters used are listed in Table 1 and are taken from references [21,26]. 

**Figure 2:**
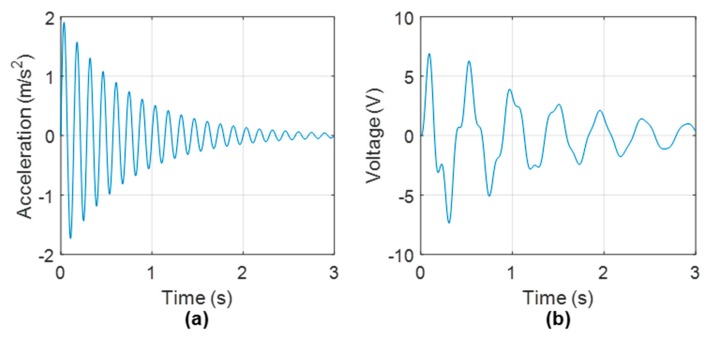
(**a**) Harvester base excitation (${\ddot{y}}_{b}$) and (**b**) Harvester output voltage.

**Figure 3:**
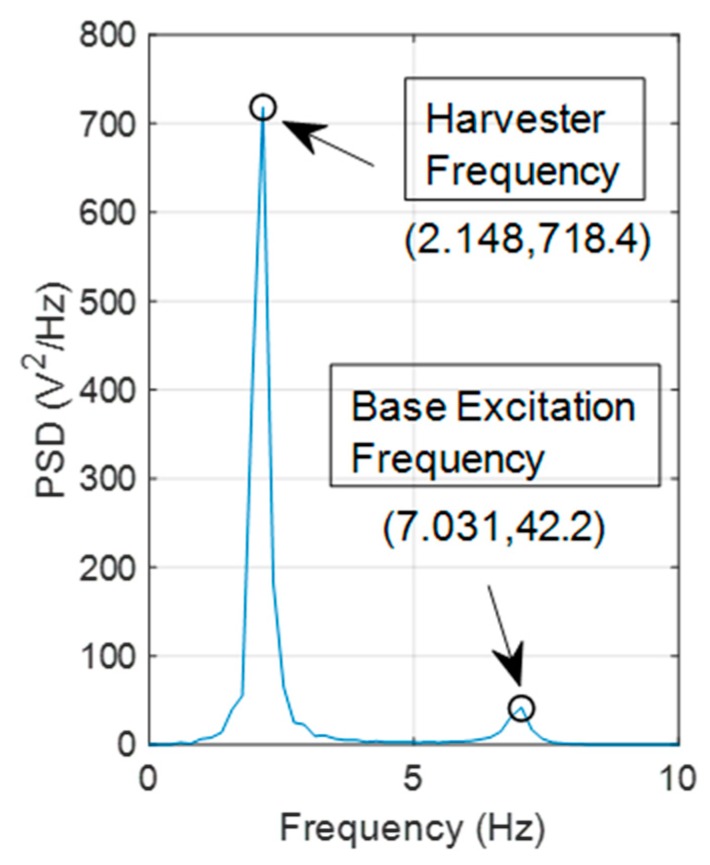
PSD of harvester voltage shown in Figure 2b.

Figure 2 shows the outputted harvester voltage generated from the input base excitation using the parameters in Table 1. Note that the initial conditions were set to zero when solving the coupled ODE’s described in Equations (1) and (2), which were solved in the MATLAB programming environment. Figure 3 shows a Power Spectral Density (PSD) of the harvester voltage shown in Figure 2b. Two peaks at 2.148 Hz and 7.031 Hz, which are corresponding to the harvester natural frequency and base excitation frequency respectively, are clearly visible. Note that the frequency resolution of the PSD is ± 0.196 Hz, which explains the slight inaccuracies to the true harvester and base excitation natural frequencies of 2.08 Hz and 7 Hz, respectively. The aim of this work is to detect structural frequencies from free vibration harvester voltage and subsequently monitor shifts in these frequencies due to the presence of bridge scour. 

## 3. Numerical Bridge Model and Frequency Changes Due to Scour

A finite element model is first created to show how scour is modelled and how it affects the frequencies of the system. The model represents a scaled bridge with four simple spans and each pier is assumed to rest on a shallow pad foundation with underlying stiffness. The stiffness of the pad foundation is based on assumptions of soil type and pad dimensions and a scaling criterion is adopted to have an appropriate stiffness value for a laboratory-scale model. 

### 3.1. Scaled Bridge with Four Simple Spans

**Figure 4:**
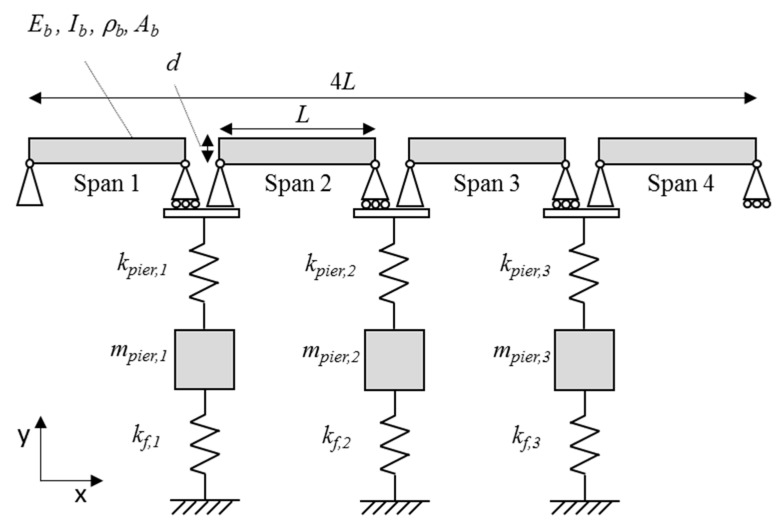
Numerical model schematic.

Figure 4 shows a schematic of the system which represents a bridge with multiple spans having pinned connections between spans (i.e., each span is simply supported). There are four spans of length L and each is modelled as an Euler-Bernoulli beam, whose mass and stiffness matrices are available in reference [27]. The beams are joined with a nodal hinge and there is a supporting pier at each joint. Each pier is modelled as a single degree of freedom (DOF) sprung-mass in the vertical direction with mass and stiffness of *k*_pier_ and *m*_pier_ respectively and each rests on a spring of stiffness, $k_{f}$, which represents the vertical stiffness provided by a shallow pad foundation. The start and ends of the bridge are assumed to be supported by undeformable abutments, which are modelled using pinned and roller supports. Hence, there are three piers supported on springs in this case. The spring supports cause an interaction to occur between spans—for example, an impulse force applied in the midspan of Span 1 (see Figure 4) causes dynamic displacements in the whole structure, whereas a static loading applied in the same place only causes displacements in the structure between the start of Span 1 and the end of Span 2.

The parameters selected in this numerical section are in fact the experimental parameters used later in the paper. This is done so that comparisons can be made between the numerical and experimental models. In selecting an appropriate value of $k_{f}$ in the scaled model, a scaling criterion is applied which checks its compliance with a full-scale dimension case. Here, a static scaling criterion is used. The ratio of the midspan deflection of a simply supported beam due to a unit static load at the centre and the deflection of a pier due to a unit static load immediately overhead, is kept constant between the full-scale and scaled down case. The stiffness of the pier, $k_{pier}$, is assumed to be infinitely stiff compared to the value of $k_{f}$ in this criterion—i.e., the equivalent stiffness of the two springs in series is assumed to be $k_{f}$. In the numerical model, the stiffness of $k_{pier}$ is selected by multiplying the value of $k_{f}$ by 104 (i.e., an arbitrary large value).

The scaling criterion is now described on a mathematical basis. The midspan deflection of a simply supported beam due to a unit load at the centre may be represented by:

(5) $$\delta_{mid}=\frac{L^{3}}{48EI}$$

where $\delta_{mid}$ is the beam mid-span deflection, L is the beam length, E is the Young’s Modulus and I is the second moment of area. The deflection of a pier, $\delta_{pier}$, due to an applied unit load directly overhead, is simply the reciprocal of the underlying foundation stiffness (i.e., 1/$k_{f}$). By maintaining a constant ratio of $\delta_{mid}$ to $\delta_{pier}$ between a full-scale case and a scaled down case, an equivalent full-scale value for the underlying foundation stiffness may be defined as

(6) $$k_{f,FULL}=k_{f,SCALED}\left(\frac{L_{SCALED}^{3}E_{FULL}I_{FULL}}{L_{FULL}^{3}E_{SCALED}I_{SCALED}}\right)$$

where subscripts FULL and SCALED refer to the full-scale case and scaled down case respectively. 

Table 2 shows the parameters used in the scaled-down model. Note, the same parameters are used for each pier and its underlying foundation stiffness. The scaling criterion defined in Equation (6) can now be used to check the validity of the $k_{f}$ value. By taking a 4 m wide single-track railway bridge as a benchmark for the full-scale case, values of $E_{FULL},$ $I_{FULL}$ and $L_{FULL}$ are assumed to be $35\;\times \;10^{6}{\;\mathrm{kNm}}^{-2}$, $0.33{\;m}^{4}$ and 20 m, respectively. By applying Equation (6), $k_{\left(f,FULL\right)}$ can be calculated to be $2.34\;\times \;10^{3}{\;\mathrm{kNm}}^{-1}$. To check this value against a geotechnical benchmark case, a shallow pad foundation of length 4 m and width 2 m is considered. By applying the approach used in reference [28], and taking reference values of sand shear modulus from reference [29], a shallow pad foundation of these dimensions has an underlying stiffness of $1.72\;\times \;10^{3}{\;\mathrm{kN}\;m}^{-1}$ for a loose sand stiffness profile and $3.44\;\times \;10^{3}{\;\mathrm{kN}\;m}^{-1}$ for a medium-dense sand stiffness profile. The value of *k*_f,FULL_ lies in this range. Hence, *k*_f,SCALED_ may be understood to represent the underlying foundation stiffness of a shallow pad foundation lying on a loose to medium-dense uniform sand deposit and is therefore an appropriate value to use. 

### 3.2. Scour Modelling and Frequency Changes of System Due to Scour

**Figure 5:**
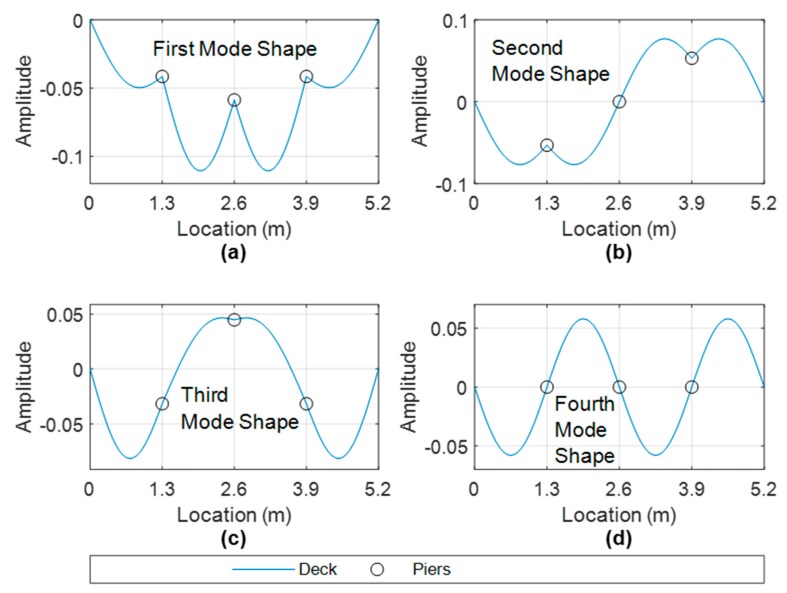
First four mode shapes of the numerical system (for healthy case)—(**a**) 9.66 Hz, (**b**) 10.55 Hz, (**c**) 12.08 Hz, (**d**) 13.85 Hz.

In this study, scour is modelled as a reduction in stiffness of the spring $k_{f}$ (Figure 4). This reduction represents the stiffness loss incurred as a result of scour. Table 3 shows the first four frequencies of the bridge system depicted in Figure 4 for the healthy case and also for scour scenarios of 24.5% stiffness loss and 44.9% stiffness loss at the central pier, Pier 2. Decreases in the frequency magnitude of Mode 1 (9.66 Hz) and Mode 3 (12.09 Hz) are apparent and these changes decrease further with a greater reduction in spring stiffness. The frequency magnitude of Mode 1 is also showing a greater change than Mode 3 with a percentage frequency change of 5.3 % versus 2.8% for the 24.5 % stiffness loss case. Mode 2 (10.55 Hz) and Mode 4 (13.85 Hz) are unaffected by the stiffness loss at *k*_f,2_. This is explained by examination of the corresponding mode shapes of the system shown in Figure 5, which are obtained using the system mass and stiffness matrices to solve the eigenproblem [30]. The mode shape values at Pier 2 (which is at the 2.6 m point) have a magnitude of zero for the 10.55 Hz and 13.85 Hz modes. Hence, these modes would not be expected to change due to a stiffness loss at this point. It is worth noting that in the generation of the mode shapes and frequencies in this section, the value of the cross-sectional area, $A_{b}$, and second moment of area, $I_{b}$, used is $2549{\;\mathrm{mm}}^{2}$ and $21.67\;\times \;10^{3}{\;\mathrm{mm}}^{4}$ respectively. These values are used to take into account two steel tracks of 8 mm side square cross-section which were present on the beam in the experimental setup described in the next section. 

## 4. Experimental Description

This section describes the experimental setup which was located in a laboratory at Kyoto University in Japan.

### 4.1. Experimental Bridge

**Figure 6:**
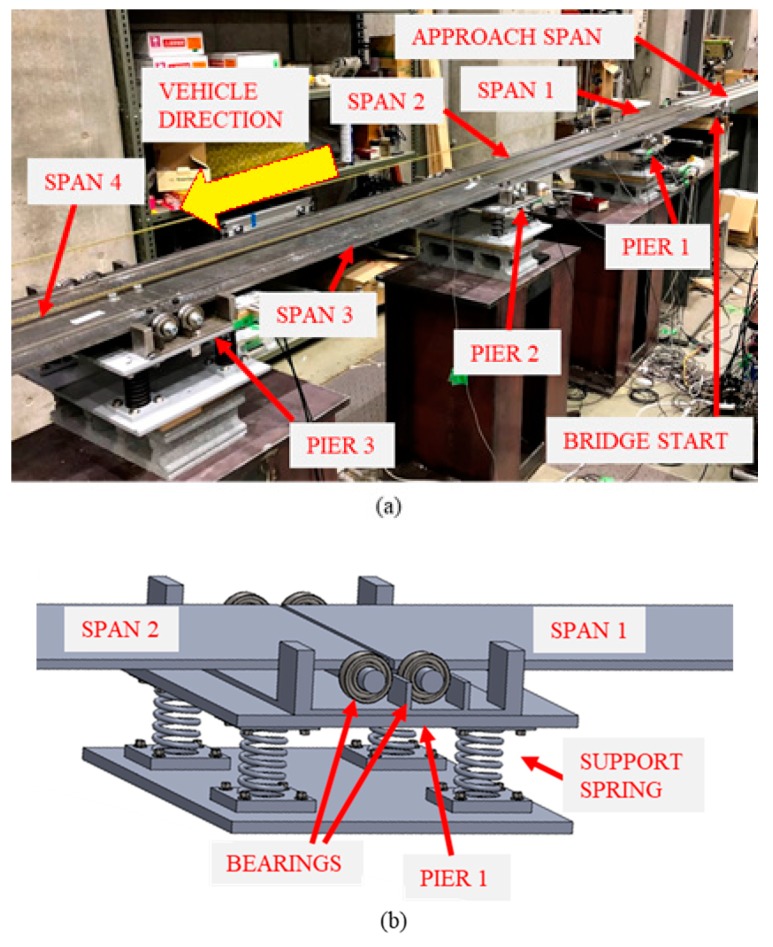
Laboratory-scaled bridge (**a**) complete bridge (**b**) schematic of Pier 1 supported on springs.

**Figure 7:**
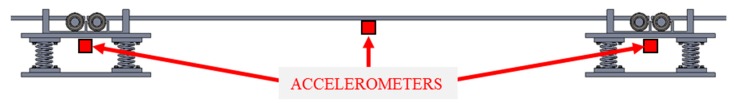
Accelerometer locations.

Figure 6a shows the scaled bridge with four simply supported spans that was used in the experiments. It contains three piers that were supported on springs (Figure 6b) while the start and ends of the bridge were supported such that they cannot deflect. Four parallel springs were used at each pier to provide vertical stability and bearings were used at either end of each span to create pinned and roller supports (Figure 6b). The properties of the experimental bridge are those specified in Section 3 and the width of each span was 300 mm. Here, each support spring for the healthy scenario had a stiffness of 49 N mm^−1^ giving an equivalent stiffness of $196{\;N\;\mathrm{mm}}^{-1}$ at each support. The stiffness of the springs was calculated from load-displacement tests. To model scour, four springs of stiffness $37{\;N\;\mathrm{mm}}^{-1}$ and four springs of stiffness $27{\;N\;\mathrm{mm}}^{-1}$ were available to replace the $49{\;N\;\mathrm{mm}}^{-1}$ springs. This was to model scour scenarios of 24.5% and 44.9% stiffness loss respectively. Three scour scenarios were investigated—24.5% stiffness loss at Pier 2, 44.9% stiffness loss at Pier 2 and 24.5% stiffness loss at Pier 3. As part of the analysis, acceleration data was also used, and accelerometers were installed at each pier and at the bridge midspans in the locations shown in Figure 7. A total of seven accelerometers were used (i.e., three pier locations and four midspan locations). There were also optical sensors installed at the start and ends of the bridge. This enabled the detection of an arriving/exiting vehicle axle.

### 4.2. Energy Harvesting Device (EHD)

**Figure 8:**
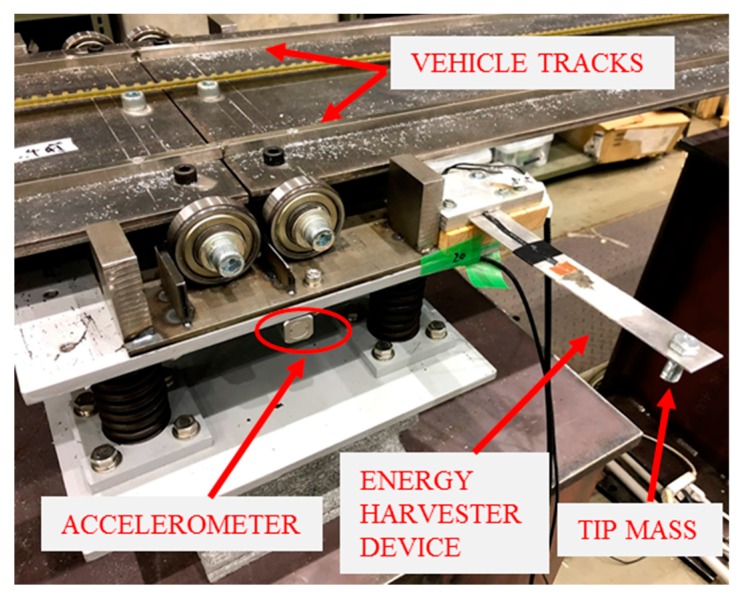
EHD installed on bridge pier.

**Figure 9:**
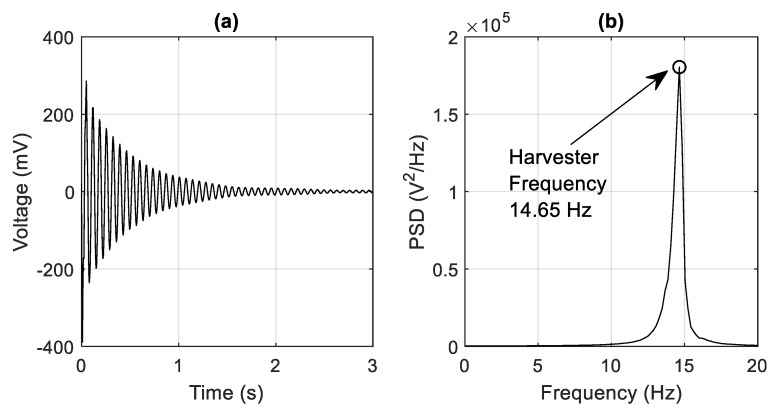
(**a**) Harvester voltage after impulse load test (**b**) PSD of harvester voltage from load impulse test.

Figure 8 shows the location of the EHD which was installed at the central pier (i.e., Pier 2). There was also an accelerometer at the pier so that the energy harvesting signal could be compared against the corresponding base excitation accelerations. The piezoelectric material used in the construction of the EHD was PolyVinyliDene Fluoride (PVDF). PVDF has properties of having good flexibility and mechanical strength, making it a desirable material to use for this application [23]. The PVDF material used had a thickness of 52 μm and a modulus of elasticity, E, and piezoelectric constant, e_31_, of 8.3 GPa and $0.1826{\;\mathrm{Cm}}^{-2}$ respectively. The PVDF also possessed two silver electrodes which allowed the output voltage to be recorded by attaching two solid core wires to them using copper conductive adhesive tape. An adhesive epoxy was then used to mechanically bond the piezoelectric harvester to the surface of an aluminium substrate. The aluminium substrate had a length, width and thickness of 177.5 mm, 25.6 mm and 1.2 mm respectively, and a modulus of elasticity of 69 GPa. There was also an attached tip mass of 19.1 g. The natural frequency of the cantilever has been determined to be 14.65 Hz. This has been obtained by carrying out an impulse load response test on the EHD and examining the open circuit response voltage (Figure 9a) in the frequency domain. Figure 9b shows a PSD of the output voltage from the impulse load test with the harvester natural frequency correlating to a peak at 14.65 Hz.

### 4.3. Vehicle

**Figure 10:**
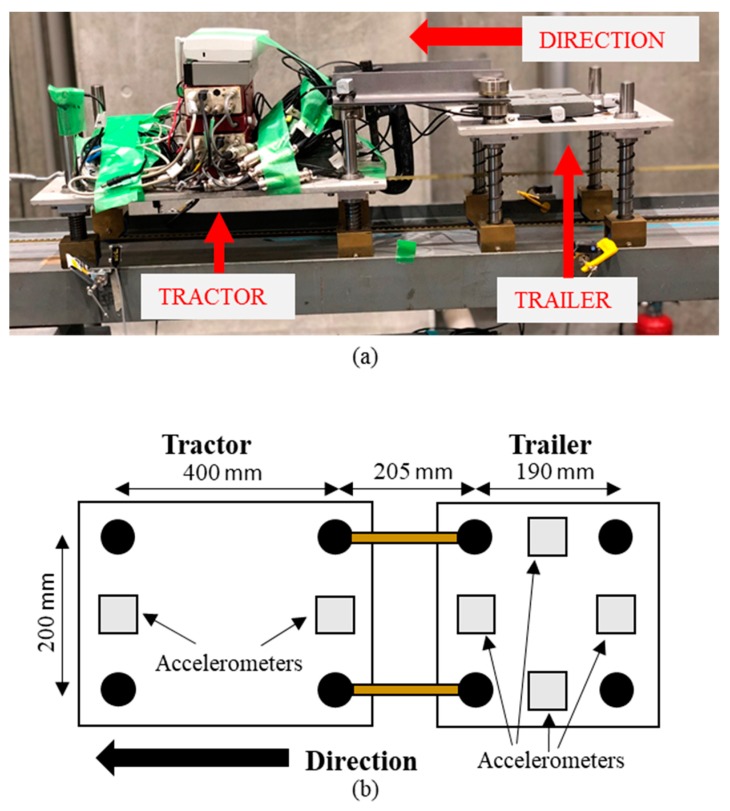
(**a**) Experimental vehicle consisting of two-axle tractor towing a two-axle trailer, (**b**) Plan view showing vehicle dimensions.

Figure 10a shows the experimental vehicle that was used as a bridge exciter. It comprised of a tractor and a trailer. It was kept on the bridge by two 8 mm square cross-section steel tracks attached to the beam (Figure 8). The tractor and trailer each had a main body consisting of a steel plate supported by four sprung wheels. The two front tractor axle wheels each had a suspension spring of stiffness $1533{\;N\;m}^{-1}$ while the two rear tractor axle wheels each had a suspension spring of stiffness $1753{\;N\;m}^{-1}$. The trailer had four suspension springs (one for each wheel) with each spring having a stiffness of $8464{\;N\;m}^{-1}$. There was a gap of 205 mm between the rear tractor axle and front trailer axle and the tractor and trailer had front-to-rear axle spacings of 400 mm and 190 mm respectively (Figure 10b). The vehicle speeds used in the experiment were 1.2 m/s and 1.26 m/s and the tractor and trailer had masses of 24.3 kg and 13.7 kg, respectively. Accelerometers were also installed on the vehicle in the locations shown in Figure 10b to aid with the identification of the vehicle natural frequencies. Using free vibration acceleration data in conjunction with a Frequency Domain Decomposition (FDD) algorithm [31], the bounce and pitch frequencies of the tractor were identified as 3.1 Hz and 4.7 Hz respectively, while the trailer had frequencies of 6.6 Hz (bounce) and 3.5 Hz (pitch). 

## 5. Experimental Results

The frequency changes due to scour are examined in this section and the frequencies obtained from free vibration acceleration data and harvester voltages are compared. Free vibration data is used as it is generally easier to extract the structural frequencies from as opposed to the forced vibration stage [8]. In order to supplement the analysis, the mode shapes of the structure are also extracted using acceleration data as input to a Frequency Domain Decomposition (FDD) algorithm [31]. 

### 5.1. Mode Shapes of Structure Extracted from Acceleration Data

**Figure 11:**
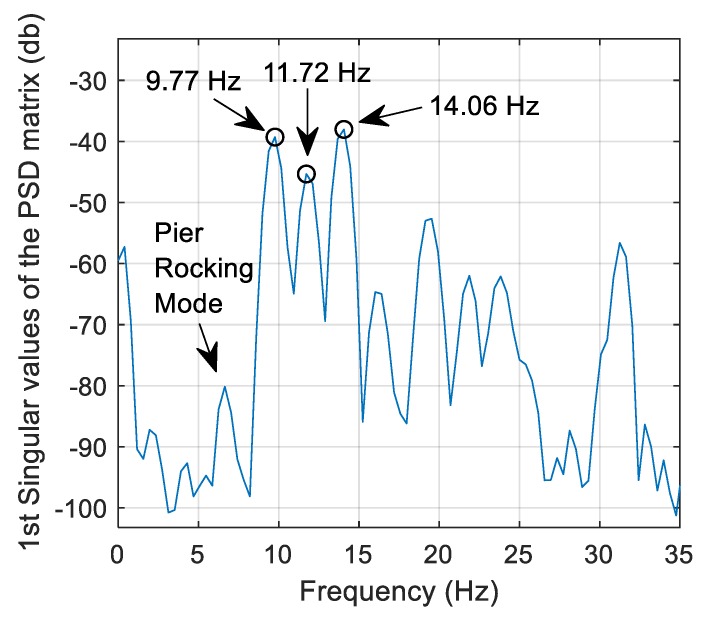
FDD frequency picking procedure.

Figure 11 shows the first part of the FDD analysis which involves selecting peaks from singular values of the power spectral density (PSD) matrix. Here, 6 s of free vibration acceleration data from the experimental vehicle crossing at 1.26 m/s is used as the input to the FDD algorithm. Data from seven accelerometers are used in this case with the accelerometer positions described previously. Here, the first three significant peaks are selected in Figure 11. These are, 9.77 Hz, 11.72 Hz and 14.06 Hz, and these peaks should correspond to the first few natural frequencies of the structure. Before the peak of 9.77 Hz, a smaller peak is also visible at 6.64 Hz which, upon further investigation, correlated to a pier rocking mode. This was clarified by installing accelerometers on both sides of the pier—the mode shape obtained at this frequency showed a clear rocking motion. 

**Figure 12:**
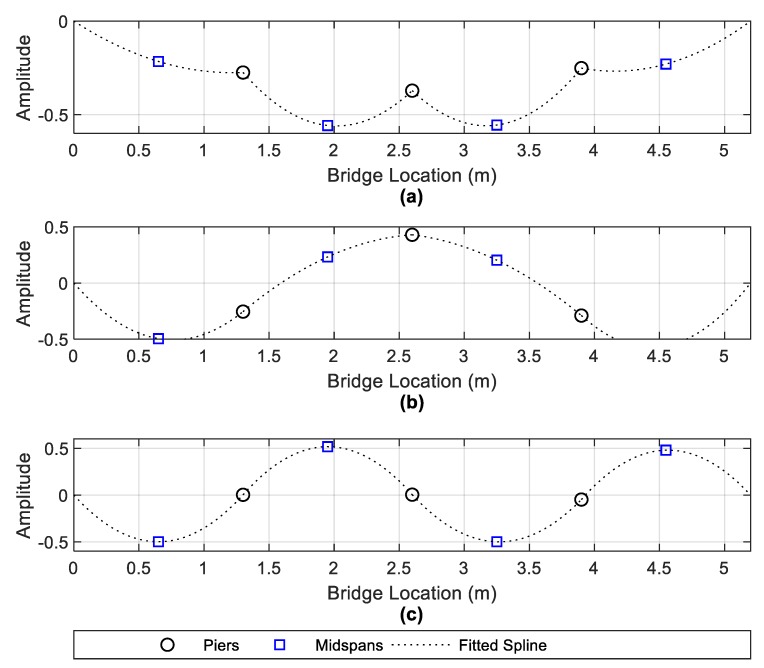
Mode shapes obtained from FDD algorithm—(**a**) 9.77 Hz mode, (**b**) 11.72 Hz mode and (**c**) 14.06 Hz mode.

Figure 12 shows the outputted mode shapes from the FDD algorithm corresponding to the frequency selections shown in Figure 11. A fitted spline has been added to the seven points to help visualise the complete mode shape. The mode shapes in Figure 12 correlate quite well with the numerical mode shapes depicted in Section 3. By comparing Figure 5 and Figure 12, it is clear that the numerical modes of 9.66 Hz, 12.09 Hz and 13.85 Hz are correlating with the experimentally derived modes of 9.77 Hz, 11.72 Hz and 14.06 Hz respectively. The second numerical mode of 10.55—(Figure 5b) has not been sufficiently excited to show in the experimental FDD frequency picking procedure.

The derivation of the mode shapes in Figure 12 is beneficial for the scour detection procedure. By examining the mode shapes, one can say whether the frequency would be expected to change due to scour. For example, in the 9.77 Hz and 11.72 Hz modes (Figure 12a,b), each of the three piers have a significant modal amplitude. The importance of this is that the frequencies of these modes are then liable to change due to scour (i.e., loss of stiffness) at a given pier. Conversely, in the 14.06 Hz mode (Figure 12c), the piers have negligible modal amplitude and hence would have little or no sensitivity to scour at a pier. In fact, this particular modal frequency is equivalent to the first natural frequency of a simply supported beam (of length 1.3 m). The frequency changes in the 9.77 Hz and 11.72 Hz mode due to scour are therefore the focus of this section, with the effectiveness of a single accelerometer at the central bridge pier being compared against an energy harvesting device at the same location. 

### 5.2. Acceleration Data Versus Energy Harvesting Voltage to Detect Bridge Frequencies 

**Figure 13:**
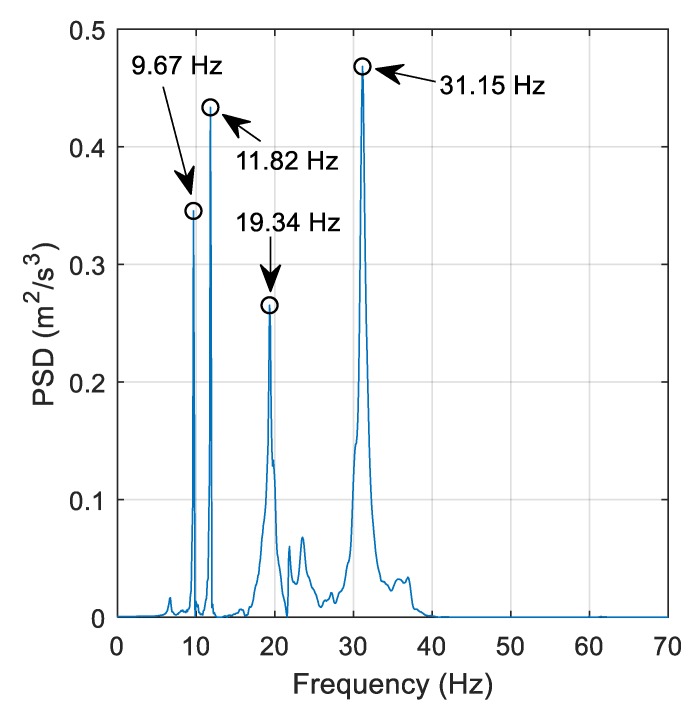
PSD of free-vibration acceleration data.

Figure 13 shows a PSD of 6 s of free vibration acceleration data where a vehicle travelling at 1.26 m/s was the source of bridge excitation. The data is obtained from an accelerometer at the central pier. A number of structural frequencies have been detected, including the first two modes shown in Figure 12. Note, the frequency resolution in Figure 13 is ±0.1 Hz, so there may be small discrepancies in the frequency peak obtained. There are also some higher modes being excited with noticeable frequency peaks at 19.34 Hz and 31.15 Hz.

**Figure 14:**
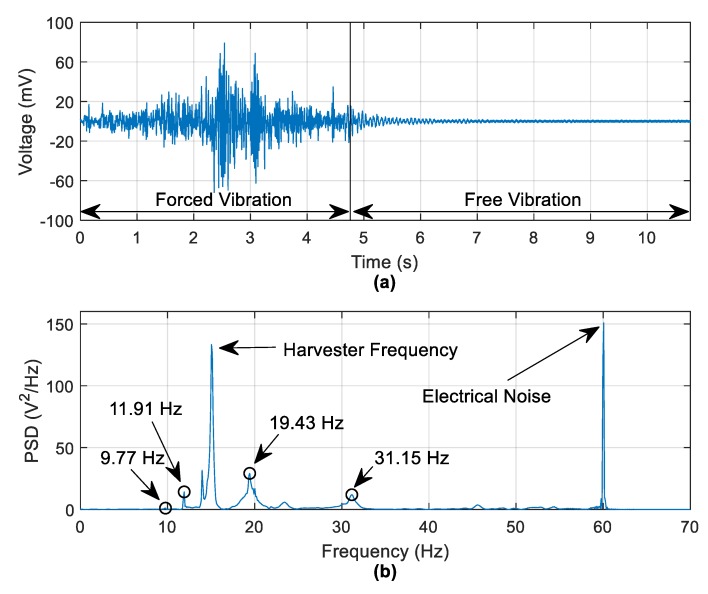
Energy harvester voltage data—(**a**) Harvester voltage, (**b**) PSD of free-vibration voltage data.

Figure 14 shows the harvester data obtained from the same vehicle run. Figure 14a shows that the highest harvester voltage is obtained during the forced vibration stage, which is expected. The focus here is on the 6 s of free vibration harvester voltage. Figure 14b shows that the same frequencies observed in Figure 13 are also present in the frequency domain of the free-vibration harvester voltage with the small frequency differences between Figure 13 and Figure 14 being due to the frequency resolution of the plots. The 9.77 Hz frequency is not visible in the current scale in Figure 14b but can be seen on a magnified scale. Figure 14b possesses two distinct frequency peaks that are not seen in Figure 13. These are, the harvester natural frequency and a 60 Hz frequency as a result of electrical noise, as the electrical system in the Kyoto region operates at 60 Hz [32]. In spite of the electrical noise, the detection of the structural frequencies from the harvesting voltage is promising. 

### 5.3. Frequency Changes Due to Scour—Accelerometer Versus EHD

**Figure 15:**
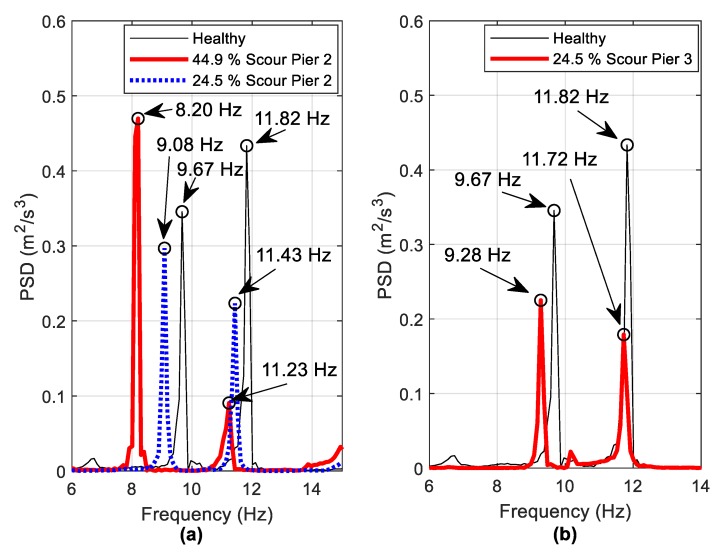
Frequency changes derived from acceleration data (**a**) Scour at Pier 2, (**b**) Scour at Pier 3.

Three scour scenarios were investigated to see how the detected frequencies change as a result. These include 24.5% and 44.9% stiffness losses at Pier 2 and 24.5% stiffness loss at Pier 3. Figure 15 focuses on the two identified scour-sensitive modes identified earlier. Again, the acceleration data from the central pier is used. It is clear that frequency shifts in both modes are detectable. Figure 15b shows that the frequency changes due to an off-centre pier are lower than for the same stiffness loss due to scour at the central pier (i.e., 0.39 Hz versus 0.59 Hz for the first mode). This is again explained by examining the mode shapes shown in Figure 12, where it is seen that the mode shape amplitude of the central pier is greater than for off-centre piers. Also, as the detected frequencies are for global modes of the bridge, the accelerometer does not have to be installed at the scoured pier to detect changes, as Figure 15b demonstrates. 

**Figure 16:**
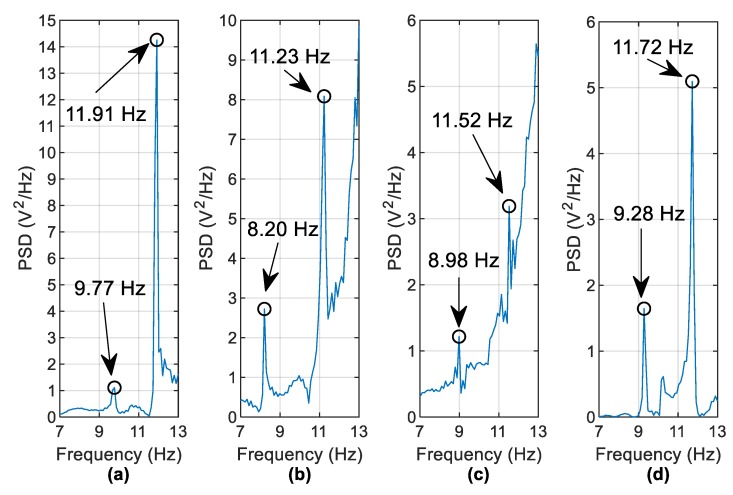
Frequencies observed from PSD of harvester voltage (**a**) Healthy case (**b**) 44.9% scour at pier 2 (**c**) 24.5% scour at pier 2 (**d**) 24.5% scour at pier 3.

**Figure 17:**
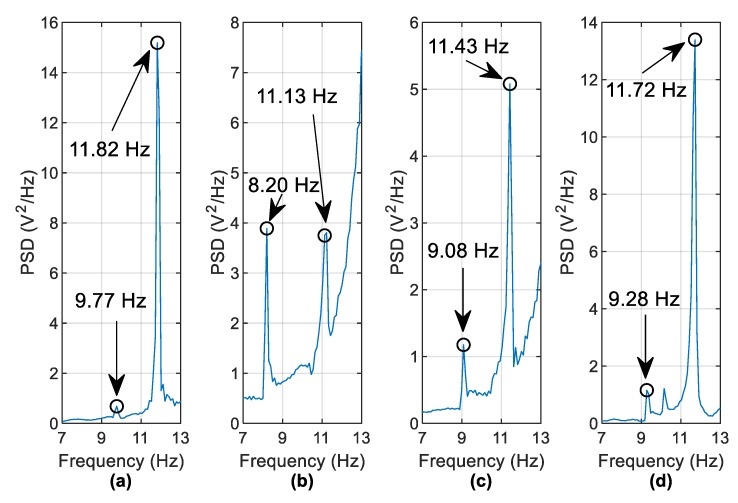
Averaged PSDs of 10 runs (**a**) Healthy case (**b**) 44.9% scour at pier 2 (**c**) 24.5% scour at pier 2 (**d**) 24.5% scour at pier 3.

Figure 16 shows a PSD of the harvester free-vibration data for the same scenarios. The identified frequencies (to within the frequency resolution of 0.1 Hz) in Figure 15 are detectable in the harvester voltage frequency domain (Figure 16) but the peaks are a lot less distinct. For example, the 8.98 Hz and 11.52 Hz frequencies shown in Figure 16c are not very clear to identify. In an effort to improve this, it is found that the use of harvester data from multiple vehicle runs is beneficial. Instead of examining the PSD of a single vehicle run, the averaged PSD (using the same frequency bins for each run) may be examined. Figure 17 averages the PSDs of free vibration voltages from five vehicles crossing at 1.26 m/s  and five vehicles crossing at 1.2 m/s (i.e., ten runs are averaged). The vehicle masses were kept constant during the experiment. The frequency identification process is clearer as a result of the averaging process, with the most noticeable improvements seen by comparing Figure 16c and Figure 17c. 

Another mode of vibration is also visible in Figure 17d between the 9.28 Hz and 11.72 Hz peaks and occurs at a frequency of 10.16 Hz. From the numerical analysis shown in Figure 5, it is known that there is a mode existing between these two modes (which occurs at 10.55 Hz in the numerical model). The absence of this knowledge is not a major problem in a real case, as, in general, only frequency decreases are of interest. Scour may be indicated by examining the frequency peak closest to the mode of interest that is either equal to or less than that of the healthy case. Here, it is clear that the 10.16 Hz frequency is from a different mode because there are two other peaks correlating to the reduction of the two modal frequencies of interest from the healthy case. 

### 5.4. Removal of Harmonics and Operational Noise for the Experimental Study

**Figure 18:**
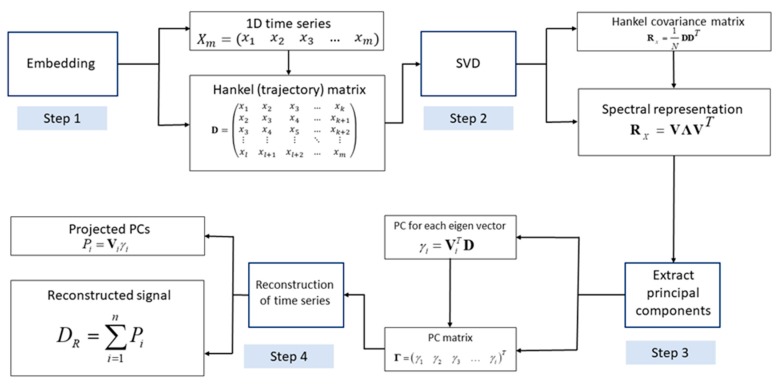
Basic steps of the SSA algorithm.

The presence of noise is inevitable during physical processes due to random variations in entities such as voltage and current. In most applications, it becomes necessary to remove the noise component prior to subsequent data analysis. Thus, the use of singular spectrum analysis (SSA) as a filter bank in noise reduction is well documented in the literature [33,34]. The method represents a potential alternative to the available filtering techniques based on eigen-decomposition on the Hankel covariance matrix obtained from a single channel of output data [34]. The resulting time series can be reconstructed by using the principal components that correspond to the actual signal constituents, thereby leaving the random (or noise) component behind. The basic steps of the algorithm are illustrated in Figure 18. 

**Figure 19:**
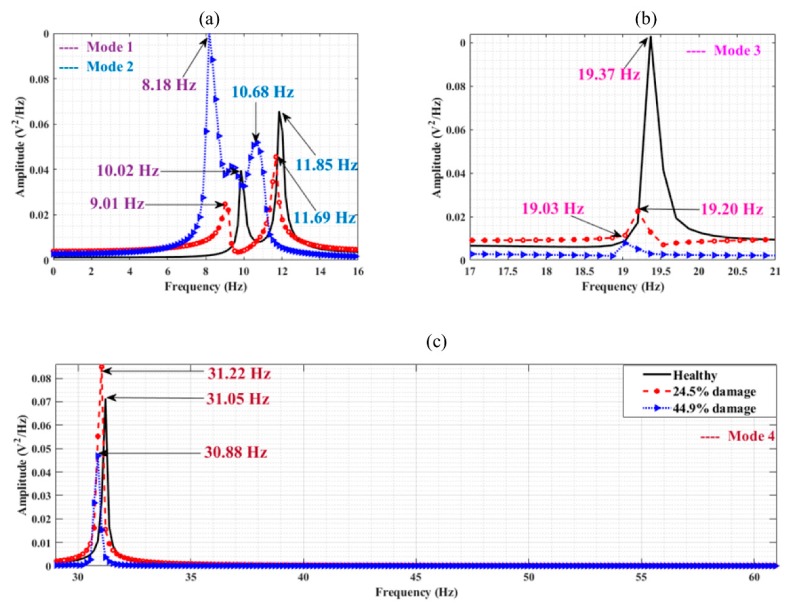
Frequencies observed from PSD of harvester voltage, (**a**) first and second vibratory modes, (**b**) third mode (filtered harvester frequency), (**c**) fourth mode (filtered electrical noise), corresponding to the test cases.

The frequency plots obtained from the experimental data indicate the presence of certain unwanted components such as the electrical noise (at 60 Hz) generated during experimentation in the Kyoto region. As the presence of noise components during experimental trials is inevitable, it becomes necessary to eliminate it using filter banks such as SSA. The present section aims at utilising the filter bank property of SSA to eradicate the noise and harmonic components of the signal. The method represents a viable alternative to the available windowed filtering techniques by providing a set of transformed reduced order responses obtained from a single sensor input, which can be further utilised for damage detection purposes [34]. The energy harvester voltage data obtained from the channel at Pier 2 is considered. The acquired signals corresponding to the healthy and the damaged states of the system are provided as inputs to the SSA algorithm, sequentially. The method proceeds by constructing a Hankel covariance matrix and selecting the first few principal components from the extracted singular values. It can be observed from Figure 19 that the algorithm is effective in removing both the harmonic load and the operational noise component that were inevitably generated during experimentation. Figure 19a indicates the frequency content for the first two vibratory modes corresponding to the test cases. The filter bank property of SSA can be precisely observed from both Figure 19b,c where the unwanted frequency components (viz., harvester frequency around 15 Hz and electrical frequency at 60 Hz) are removed by reconstructing the signal constituents. Through the frequency shifts, the figure provides elementary information regarding the healthy and damaged states of the pier, which emulates a physical scenario of a scour process.

**Figure 20:**
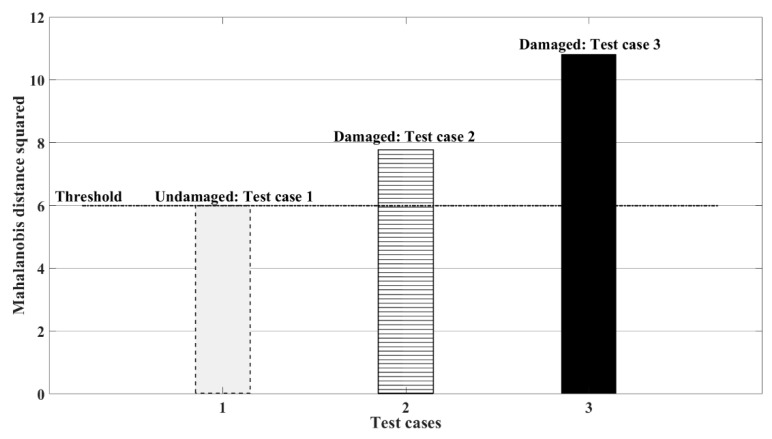
Mahalanobis distance as an indicator of damage to the experimental setup; test case 1: Healthy case, test case 2: 24.5% scour at Pier 2, test case 3: 44.9% scour at Pier 2.

The use of frequency shifts as a preliminary indicator of damage is well reported in the literature [34,35]. SSA provides reduced order responses corresponding to the different states of the system, from which a clear indication of damage is evident, as illustrated in Figure 19. To further confirm the findings, the authors have used Mahalanobis distance to distinguish the different states of the system [36]. The ensemble mean of the datasets is first considered to train the samples for live testing collected at 200 Hz. Appropriate auto-regressive (AR) models are fit to the time series, from which a threshold is generated based on the assumption that the underlying variables are normally distributed. This implies that the distribution of the square of the Mahalanobis distance will be chi-squared with degrees of freedom equal to the number of AR coefficients [36]. From Figure 20, it can be clearly observed that while the first dataset corresponding to the undamaged case is well within the threshold, the square of the Mahalanobis distance for the damage cases lie beyond the threshold, thereby indicating damage to the system. The evidence from this study suggests the use of SSA as an efficient filter bank approach that can be utilised for subsequent damage detection of practical case studies. 

### 5.5. Discussion

The detection of the structural frequencies in the harvester voltage and the subsequent change detected due to scour has shown that an energy harvesting device has the potential to be used as a bridge scour monitoring tool. Furthermore, the stringent constraint of sensor instrumentation at each pier is eliminated through the use of SSA where a single harvesting device can be used to detect a frequency change due to scour. The best results have been acquired through the averaging of multiple harvester voltage data in the frequency domain and filtered using an appropriate SSA model. In order for an energy harvester to be used as a stand-alone scour monitoring device (that does not necessitate the use of a power supply), a modal analysis of the bridge using multiple accelerometers at the time of harvester installation would be beneficial to the scour-monitoring procedure. This would entail the temporary installation of a power source for the accelerometers and data acquisition system until sufficient acceleration data can be collected to derive the mode shapes and frequencies associated with the bridge structure. The identification of potential scour sensitive modes then allows for the monitoring of their associated frequencies detected from the harvester voltage. This approach of using energy harvesters as structural health monitors can be relevant for long span bridges [37], as has been observed before for open cracks [38] and pipelines [39]. Real-time monitoring and detection techniques can be integrated with this approach, especially where the techniques have demonstrated lower levels of detections of stiffness loss (of the order of 10–15% reduction) [34,40]. Small changes in boundary conditions brought about by scour can bring significant stiffness reductions [41] and laboratory scale testing has scaling aspects to be considered [42]. The scaled testing is also important in this regard since most laboratory based testing [43] or full-scale assessments [44] come from a hydrological point of view and without an assessment of the stiffness reduction.

## 6. Conclusions

This paper proposes a bridge scour detection approach that monitors changes in bridge frequencies detected from the free-vibration voltage of a cantilever-based piezoelectric energy harvesting device (EHD) attached to the bridge. A demonstration in a laboratory-scaled bridge consisting of four simple spans has verified this. It is shown that the use of one EHD attached to a bridge pier can detect frequency shifts arising from a loss of stiffness due to scour under a support. Also, it is shown that the EHD attached to a healthy pier can detect a frequency change due to scour at another pier. This is because the frequency detected is from a global vibration mode, meaning that the location of the EHD is less of an issue. Furthermore, changes in two vibration modes due to scour have been detected from the EHD voltage. Improved results have been achieved by averaging the free-vibration voltage from different vehicle runs in the frequency domain. The harmonic loading and deterministic operational noise component arising due to electrical interference in experimental trials is removed using SSA as a filter bank. The transformed response obtained using the single channel EHD data is further utilised to distinguish between the damaged and undamaged states of the system, thereby verifying the findings from the frequency domain counterparts. It is envisaged that the method would work best with an initial modal analysis of the bridge using accelerometers, after which identified modal frequencies could be monitored in the EHD voltage frequency domain.

## Figures and Tables

**Table 1:** Harvester parameters used in the simulation [21,26].

Property	Symbol	Value	Unit
Tip mass	$m_{h}$	2.5	g
Equivalent viscous damping ratio	$\xi_{h}$	0.038	-
Stiffness	$k_{h}$	0.4286	N m^−1^
Natural Frequency	$f_{h}$	2.08	Hz
Electromechanical Coupling	θ	7.501	μC m^−1^
Capacitance of the piezoceramic material	$C_{p}$	2.866	nF
Nondimensional time constant	α	0.9	-
Resistance of energy harvester	R_l_	1000	kΩ

**Table 2:** Model properties.

Property	Symbol	Unit	Value
Span length	L	mm	1300
Beam depth	d	mm	8.07
Beam cross-sectional area	$A_{b}$	${\mathrm{mm}}^{2}$	2421
Beam second moment of area	$I_{b}$	${\mathrm{mm}}^{4}$	$13.14\;\times \;10^{3}$
Beam modulus of elasticity	$E_{b}$	${\mathrm{Nm}}^{-2}$	$2.05\;\times \;10^{11}$
Beam density	$\rho_{b}$	${\mathrm{kgm}}^{-3}$	7850
Underlying foundation stiffness	$k_{f}$	${\mathrm{Nmm}}^{-1}$	196
Pier mass	$m_{pier}$	kg	12.56
Pier stiffness	$k_{pier}$	${\mathrm{Nmm}}^{-1}$	$196\;\times \;10^{4}$

**Table 3:** System frequencies due to scour at Pier 2 (i.e., due to reduction in stiffness, *k*_f,2_).

Mode Number	Healthy	24.5% Scour	44.9% Scour
1	9.66 Hz	9.15 Hz	8.36 Hz
2	10.55 Hz	10.55 Hz	10.55 Hz
3	12.09 Hz	11.75 Hz	11.48 Hz
4	13.85 Hz	13.85 Hz	13.85 Hz
